# Plant MITEs: *miniature* transposable elements with *major* impacts

**DOI:** 10.1186/s13100-025-00375-8

**Published:** 2025-11-07

**Authors:** Abirami Soundiramourtty, Marie Mirouze

**Affiliations:** 1 University of Perpignan, Perpignan, France; 2 EMR269 MANGO (CNRS/IRD/UPVD), Laboratory of Plant Genome and Development, Perpignan, France; 3 Institut de Recherche Pour Le Développement (IRD), Laboratory of Plant Genome and Development, Perpignan, France

**Keywords:** Miniature inverted repeat transposable elements, Plants, Epigenetic, Transcription factor

## Abstract

Miniature inverted repeat transposable elements (MITEs) are short, non-autonomous transposable elements that have attracted considerable attention over the years due to their ubiquitous presence and functional roles in plant genomes. A growing body of evidence points to a complex and multifaceted interplay between MITEs and host genomes. This review aims to elucidate the diverse roles of MITEs in shaping plant genome architecture, gene expression and adaptability to environmental stresses through different molecular mechanisms such as accommodation of regulatory sequences, promotion of alternative splicing, generation of epialleles and small RNAs, and mediation of structural variation. These examples highlight the functional importance of MITEs in plant genomes and provide directions for future research.

## Introduction

Transposable elements (TEs) are mobile segments of DNA that accumulate in almost all eukaryotic genomes, accounting for up to 85–90% of the total genome in some crop plants such as maize, barley or wheat [46]. Their early reputation as 'junk' DNA or genomic parasites detrimental to the host organism is now slowly fading. Over the past few years, recent advances have shed light on how TEs can have complex interactions with genes and their potential to contribute to variation in gene expression, gene regulatory networks and evolution [1, 4, 23, 40, 69]. TEs are classified into class I (use of a RNA molecule as molecular intermediate of transposition) and class II (use of a DNA molecule as molecular intermediate of transposition) elements. Miniature Inverted Transposable Elements (MITEs) are class II elements present in eukaryotic genomes, notably in plant genomes.

### MITE discovery

MITEs were first discovered by Susan Wessler during the 1980s by studying maize waxy (*Wx*) mutants. The *waxy* locus encodes a key enzyme involved in starch synthesis and is expressed in kernels and pollen grains. Mutant kernels exhibit a waxy appearance as a direct result of changes in starch composition. Using spontaneous and induced *wx* mutant, several TE insertion polymorphisms (TIPs) were found at the waxy locus. Among them a particularly small insertion of 128 bp in the *wxB2* allele led to the discovery of a new class of transposable element named *Tourist* [6, 70]. Similarly, while investigating a *Tourist* insertion in *Sorghum bicolor* called *Tourist-Sb5* and located in the phosphoenolpyruvate carboxylase gene, a 257 bp insertion within *Tourist-Sb5* allowed for the identification of a new class of transposon called *Stowaway* [7]. Since these seminal discoveries, MITEs have been widely identified in plant and animal species. As originally identified by the Wessler laboratory, most MITEs in plant genomes are divided into two major groups: *Tourist*-like MITEs (derived from *PIF*) and *Stowaway*-like MITEs (derived from *Tc1/Mariner*). However, MITEs can also originate from other Class II TE families, such as *CACTA*, *hAT* and *Mutator*. Generally, while MITEs occupy a small fraction of plant genomes, ranging from 0.06%, 2%, 5% to 10% in papaya, maize, apple, and rice, respectively [13], they tend to be present in abundant copy numbers.

### MITE features

**Fig. 1:**
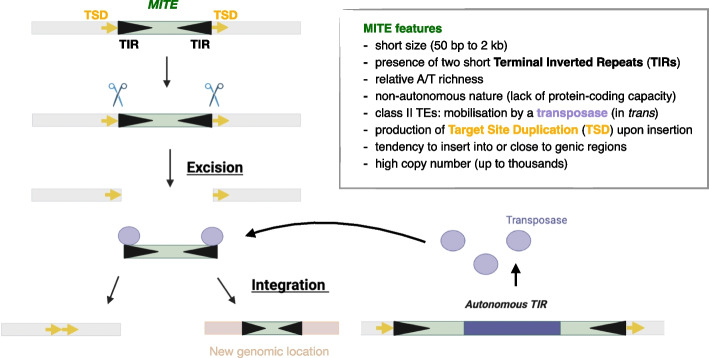
Key features of MITEs. MITEs are class II transposons with terminal inverted repeats (black arrows) flanked by TSD (target site duplication, usually AT-rich, in yellow). They mobilise by a cut-and-paste mechanism following excision from a donor site. As non-autonomous TEs, they rely on their autonomous counterparts for their transposase (circle) mediated movement and integration into a donor site. MITEs tend to be very small transposons with preferential insertion into genic regions

Although *Tourist* and *Stowaway* superfamilies do not share sequence similarity, they have striking structural similarities as they both carry terminal inverted repeats (TIRs). *Tourist* and *Stowaway* MITEs differ in their target site preference (TA or TWA for *Stowaways* and *Tourists*, respectively) reflecting the corresponding transposase preference. The transposases are encoded by autonomous TIR class II elements (see below). For *Tourist* MITEs the identified transposases come from the *PIF/Harbinger* family while for *Stowaway* the transposases belong to the *Tc1/Mariner* family. MITE insertions produce short (2-9 bp) A-T rich target site duplications (TSDs) [11]. Hundreds of MITE superfamilies have been computationally identified, such as *PIF/Harbinger* (including *Tourist*), *Tc1/Mariner* (including *Stowaway*), *hobo-Activator-Tam3* (*hAT)*, and *Mutator* [20, 21, 23, 53]. In summary, although there are no clear criteria to define a MITE, they share some features that distinguish them from other non-autonomous class II TEs, as listed in Fig. 1.

### Mode of transposition

Given their small size, MITEs lack protein coding sequences. Their TIRs can be recognized *in trans* by related transposases for mobilization [22, 36, 37, 75]. For instance the *Tourist mPing* family share sequence similarities with full-length TEs, suggesting that they originated from internal deletions of these elements. These copies retain their terminal inverted repeats (TIRs), enabling them to bind to and be mobilised by the transposases encoded by full-length elements [26]. For instance, the *PIF/Harbinger* elements require two proteins: ORF1, for DNA binding and the DDE/D containing transposase for mobilization [56, 74]. In contrast, the autonomous partner of *Stowaway* transposons was not identified until the Wessler’s lab performed excision assay in yeast. Using this assay they showed that rice transposases belonging to the *Tc1/Mariner* family could mobilize the distantly related *Stowaway* MITEs [74]. Interestingly, while autonomous copies contain an internal sequence repressing their transposition, this sequence is not present in *Stowaway* MITEs [74]. More recently, transposition assays in yeast with systematic mutation in the internal sequences of Tourist-like *mPing* and the relative autonomous copies *Ping* and *Pong* revealed multiple regions that either enhance or suppress transposition activity [56]. Modification of *mPing* TIRs severely decreased transposition frequency, suggesting a critical role of each nucleotide for efficient transposase binding [56]. Additionally, systematic mutation of the internal sequences identified several regions that also promote or inhibit transposition indicating internal regions coupled with TIRs within MITEs play pivotal role for regulating transposition rate to promote or self-limit their activity [56]. The mode of transposition of MITEs could be more complex, as suggested recently by Pulido and Casacuberta [55]. Indeed, in rice, a positive correlation between the presence of some specific MITE copies in the genome and the total copy number of their corresponding families suggests that only a few "master" copies could be responsible for significant amplification of MITE families [9].

### MITE impacts

MITEs can strongly influence gene expression by inserting preferentially near or within genes, thereby impacting their transcriptional regulation. MITEs play crucial roles in genome evolution, generating genetic diversity, and participating in gene and regulatory element rearrangements [11, 40]. Being highly abundant and polymorphic, MITEs promote diversity at the species, population and even somatic levels. This genetic diversity is shaped by evolutionary forces, such as natural selection and genetic drift, and can give rise to novel phenotypic traits and adaptations. Understanding the role of MITEs and their involvement in phenotypic variation and adaptive processes can provide valuable insights into the evolutionary dynamics of plant genomes [24, 40]. Here, we review recent striking examples of how MITEs influence plant gene expression and phenotype, highlighting their importance in genome evolution.

**Table:** 

What is the maximum MITE size? Although MITEs have been known about for decades, there is no clear consensus on their maximum size. Some authors consider large TEs to be MITEs, while others reserve the term 'MITE' for short TEs (less than 800 bp; [14]. We could not find a widely accepted definition in terms of size. For simplicity, in this review we do not apply a conservative size limit.	

#### MITEs insert near genes

Unlike many TEs, MITEs exhibit preferential insertions within gene-rich regions. This property might explain the extensive contribution of MITEs to gene regulation across various plant species. We illustrate below the landscape of MITE insertions in several plant species.

In the *Brassicaceae* family, the *Monkey King Tourist*-like MITE (with 504 full copies in *Brassica Rapa*, 55 in *Arabidopsis lyrata* and 38 in *Arabidopsis thaliana*) has most of its copies inserted within a 3 kb distance of a gene. This family exhibits high levels of TIPs within and between *Brassicaceae* species [16, 33]. In *B. napus*, 677 TIPs were identified among 4 accessions, 10 of which were associated with the expression of a nearby gene [33]. A comparative analysis of 19 accessions in *Arabidopsis thaliana* identified 343,485 MITE sequences. 5,712 MITEs insertions were found near annotated genes (in genes or < 40 bp to a gene) in the reference genome, with 8,390 insertions across all 19 accessions [29]. Euchromatic MITEs were inserted near introns or the final exons of genes, suggesting a potential regulatory role in the 3' UTR regions of these genes.

In rice, MITEs impact gene expression even more widely, being associated with 23,623 genes (58.2%) in the Nipponbare variety, either in intronic or regulatory regions. 7,887 MITE sequences are transcribed and at least 3,463 are co-transcribed with genes [42, 51]. Using 208 varieties belonging to the *Oryza sativa ssp.* indica and *Oryza sativa ssp.* japonica subspecies, 45,050 transposable elements polymorphisms (TIPs) were found, mainly corresponding to DNA transposons, especially MITEs [8]. Many genes associated with TIPs appear to be linked with rice domestication and breeding [8].

Similarly, around 18,500 polymorphic insertion sites of *Stowaway* elements were found in the genomes of 31 cultivated and wild carrot species (*Daucus carota*). Among these insertions, only two of these harboured the same element insertion in all 31 genomes. These elements were predominantly located close to genes, particularly within 2 kb regions flanking genes and in the 5′ and 3′ UTRs, suggesting a potential functional impact. Additionally, the *DcSto7b* family displayed a higher level of polymorphism in the cultivated carrot genome, suggesting that *DcSto7b* may have been active during carrot domestication [43].

#### MITEs have a functional impact on gene expression

MITEs can insert into gene promoters thus modulating their transcriptional activity. For example in rice, genome-wide analyses have shown that genes associated with MITEs tend to be expressed at significantly lower levels than genes located away from MITEs [42]. Some specific examples of functional impact are described in this section.

**Fig. 2:**
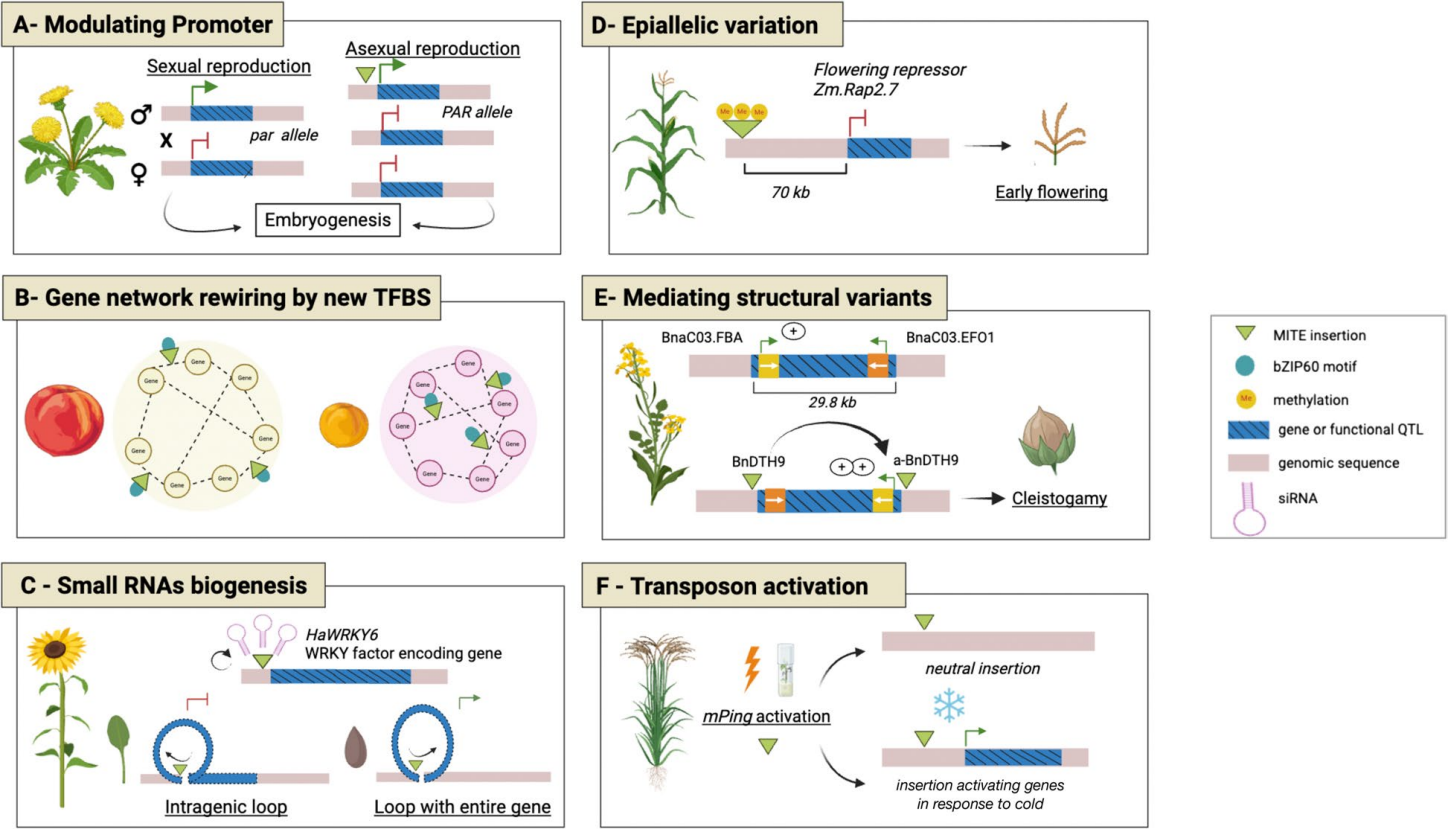
Examples of the functional impact of MITEs on plant development and adaptation. **A** In dandelions, the insertion of a MITE upstream of the *PAR* locus induces parthenogenesis in triploid plants, whereas diploid plants without the insertion reproduce via sexual reproduction. **B** A comparative analysis of *Prunus *species, such as peaches and apricots, revealed that MITE insertions harbouring *bZIP60* motifs have evolved differently, generating distinct regulatory networks (dotted lines) between genes (circles). **C** In sunflowers, a MITE insertion upstream of the *HaWRKY6* gene leads to the production of small interfering RNAs (siRNAs), resulting in tissue-specific chromatin topology. An intragenic chromatin loop represses gene expression in leaves (left), while a different alternative loop in cotyledons modulates siRNA biogenesis, thereby allowing gene expression (right). **D** In maize, the methylation of a MITE insertion located 70 kb away from a flowering repressor gene represses its expression, inducing early flowering. **E** In *Brassica napus*, the presence of two MITEs inserted in opposite directions is associated with a 29.8 kb chromosomal inversion, thereby increasing transcription of *BnaC03.FBA*, a gene containing an F-box domain. This overexpression influences petal closing behaviour and promotes cleistogamy. By contrast, in non-cleistogamous plants, *BnaC03.FBA* and *BnaC03.EFO1* are expressed (green arrow), albeit at lower levels. **F** In *Oryza sativa*, *mPing* can be activated under conditions such as tissue culture or irradiation. De novo insertion can involve rewiring of gene networks, including activation of cold-responsive genes, which are usually repressed in the absence of the insertion

In dandelions, the PAR (parthenogenesis-associated region) locus is a major quantitative trait locus (QTL) that controls both apomictic and sexual reproduction [65]. The dominant allele responsible for apomixis contains a conserved 1,335 bp *hAT* MITE insertion, which is located 110 bp upstream of the PAR locus and induces PAR gene expression. Introducing this MITE-containing PAR allele into lettuce egg cells triggered apomixis without the need for fertilisation (Fig. 2A, [65]). Interestingly, a 1,282 bp MITE insertion was also identified in apomictic variants of hawkweed 137 bp upstream of the LOP locus, which is homologous to the dandelion PAR locus. This suggests that reproductive traits have evolved in parallel in these species, possibly driven by MITEs.

Root architecture plays a pivotal role in development and in a plant's ability to withstand stressful environmental conditions. In some wheat varieties, such as Drysdale, a genome-wide association study (GWAS) identified a polymorphic 125 bp *Tc1/Mariner* MITE insertion associated with root depth during the booting stage [68]. This insertion is located within the promoter region of the TaVSR1-B gene, leading to its repression, possibly via epigenetic silencing through DNA methylation and H3K27 trimethylation. Furthermore, increased expression of this gene in transgenic lines was associated with a reduced elongation zone and an expanded differentiation zone.

A GWAS analysis in rice identified *HTG3* as a candidate gene associated with thermotolerance, particularly in the Nipponbare, ZH11 and MH63 cultivars [71]. Under heat stress conditions, HTG3 acts as a positive regulator of heat tolerance via heat shock proteins and the jasmonic acid signalling pathway. *HTG3* has two allelic forms, the most notable difference between which is the presence or absence of a 228 bp MITE insertion. The allele lacking the MITE insertion exhibited increased *HTG3* expression under normal and heat stress conditions compared to the MITE-containing allele. Hypermethylation was associated with the insertion-containing allele in the *HGT3* promoter, suggesting possible repression via methylation.

In citrus species, including sweet oranges, satsuma mandarins and lemons, somatic embryogenesis within nucellar tissues is the main cause of polyembryony. A key gene, *CitRKD1*, is principally expressed in the reproductive tissues of polyembryonic variants [61, 67]. This gene has two allelic forms: (1) *CitRKD1-mg2*, a polyembryonic allele featuring a MITE insertion upstream, and (2) *CitRKD1-mg1*, a monembryonic allele lacking it. The *CitRKD1-mg2* allele is the predominant transcript in the somatic embryogenesis of nucellar cells in polyembryonic lines. Furthermore, loss of CitRKD1 function in sweet orange resulted in loss of somatic embryogenesis, suggesting that the MITE insertion may be involved in CitRKD1 expression during somatic embryogenesis.

#### MITEs contain transcription factor binding sites

MITEs can harbour transcription factor binding sites (TFBSs) within their sequence, which have the potential to rewire new genes within pre-existing transcriptional networks when inserted near responsive genes. This can lead to changes in a cell's regulatory landscape via different mechanisms, as illustrated by the examples below.

In *Brassicaceae* species, TEs have led to the significant amplification of E2F TFBS motifs [32]. Notably, approximately 85% of these motifs are located within TEs, primarily in MITE copies belonging to the *hAT*, *PIF*/*Harbinger*, and *MULE* superfamilies, as well as in Helitrons [32]. These 10 bp motifs exhibit binding ability to E2Fa and could therefore influence gene regulatory mechanisms. However, the majority of E2F TFBSs found within MITEs are located far from genes, potentially diluting E2F transcription factors. In *Arabidopsis thaliana*, MITE-encoded E2F TFBS are found close to genes and exhibit polymorphism between different ecotypes, suggesting a possible role in adaptation [32].

Similarly, in Prunus and tomato species, MITEs have contributed to the amplification of TFBS motifs associated with several transcription factors (see Fig. 2B; [49]). The amplification of MITE copies harbouring TFBS sites for factors such as bZIP60 and PIF3 in Prunus, and TCP15/23 in tomato, has contributed to the redistribution of such motifs, playing a crucial role in the regulation of responses to biotic and abiotic stresses. For example, bZIP60 TFBS-containing MITEs were mobilised and disseminated differently among Prunus species; the resulting gene networks differ considerably, suggesting that they may have evolved differently in Prunus and peach, for instance.

A comparison of regulatory networks linking transcription factors and target genes in maize seedlings and husk tissues revealed that husk-specific enhancers appear to be enriched in *PIF/Harbinger* MITEs [19]. Furthermore, those MITEs located in husk-specific enhancers are enriched in specific transcription factor binding sites (TFBS) motifs: HB-like, MYB-like, and MADS box. Interestingly, the genes targeted by MYB-like motifs carried by these MITEs are enriched in leaf development function, pinpointing the potential role of these MITEs in shaping the spatiotemporal regulation of gene expression during maize development.

#### MITEs impact small RNAs production

MITE transcripts produced from a neighboring gene promoter can form hairpin secondary structures due to their TIRs. These dsRNAs can serve as substrates for the production of 24-nt small interfering RNAs (siRNAs). MITEs can also evolve as precursors of microRNAs (miRNAs) and circular RNAs (circRNAs), consequently affecting gene expression patterns. Recent studies showing gene regulation via the generation of siRNAs, miRNAs and circRNAs are summarised in the following section.

In the *Arabidopsis thaliana* Col-0 accession, 634 out of 885 inverted repeats (IRs) are located within 3 kb of a gene [2]. Notably, approximately 68% of these IRs likely originate from transposable elements (TEs), particularly MITEs from the MuDR family, and around 486 of the 634 IRs produce 24-nt small interfering RNAs (siRNAs). These IRs exhibit reduced DNA methylation in mutants incapable of producing 24-nt siRNAs and in those impaired in CHH methylation. Variations in IR-triggered methylation affect chromatin topology, demonstrating that methylation influences the formation of chromatin loops. Furthermore, 193 of the 634 IRs located near genes exhibit natural variation across 216 *A. thaliana* accessions, suggesting that these variations may influence chromatin structure and gene activity.

In sunflowers, a 260 bp MITE insertion located 600 bp upstream of the HaWRKY6 gene has been identified as interacting with the gene and playing a key role in modulating 3D chromatin conformation (Fig. 2C; [25]). Transcripts originating from this MITE lead to the production of 24-nt siRNAs. In leaves, a stable loop configuration involving regulatory regions and a partial HaWRKY6 gene sequence acts to repress HaWRKY6 gene expression. In contrast, cotyledons exhibit an alternative loop arrangement that encompasses the entire gene in the opposite direction to the MITE-containing promoter, thereby enabling HaWRKY6 gene expression.

In several angiosperm species, such as rice, *Brachypodium distachyon*, foxtail millet, maize, sorghum and tomato, MITEs are the main source of de novo microRNA (miRNA) synthesis [30, 53], thereby promoting diversity in their miRNA repertoire. Specifically, MITEs from the *Mutator* superfamily in dicots and the *Tc1/Mariner* and *PIF/Harbinger* superfamilies in monocots contribute significantly to MITE-miRNA production. The size and copy number of these MITE superfamilies in host genomes appear to be key factors in determining their potential role in miRNA biogenesis. MITE copies that produce miRNAs are enriched in pseudogenes and intronic regions compared to other MITE copies. The genes targeted by MITE-miRNAs are enriched in functions associated with responses to environmental stimuli, particularly temperature-related processes, which suggests that MITE-miRNAs play a role in plant adaptation to varying environmental conditions.

#### MITEs impact circular RNAs production

Circular RNAs (circRNAs) are a class of non-coding RNAs that form continuous loops, Linking the 5' and 3' ends. Identified across all eukaryotic species, they impact gene expression [77]. MITEs (as other TEs) contribute to circRNA production in plants [77].

For example, in *Populus tomentosa*, MITEs, particularly those belonging to the *hAT* and *PIF/Harbinger* superfamilies, are enriched in circRNA-producing regions and correlate with circRNA abundance levels. Furthermore, Circ_0003418 expression levels are associated with the presence of SNPs within the flanking MITE [63]. Transgenic constructs containing different configurations of Circ_0003418 and Circ_0000408, along with flanking MITEs, demonstrated that altering these elements significantly decreases transcript production, suggesting a potential *cis*-regulatory role in circRNA regulation.

#### MITEs can trigger DNA methylation

TEs attract heterochromatic epigenetic marks. Thus, MITEs can induce alterations in epigenetic patterns in gene regions, which can potentially result in modulation of gene expression dynamics. For instance, the methylation of an inserted copy can spread to nearby genes, repressing them. This section presents some examples illustrating this mechanism.

In maize, the Vegetative to Generative Transit 1 (Vgt1) locus is a well-known major QTL for regulating flowering time [58]. Vgt1 was shown to be a *cis*-regulator of the ZmRap2.7 gene, a well-known flowering repressor located 70 kb away from Vgt1 [58]. A *PIF/Harbinger* MITE insertion at the Vgt1 locus is associated with early flowering traits (see Fig. 2D,[10]). Remarkably, the MITE insertion is associated with local hypermethylation, which impacts the transcription of the ZmRap2.7 gene, leading to reduced expression and early flowering. Allele-specific methylation has been correlated with the differential transcriptional activity of ZmRap2.7*.* This detailed example illustrates the pivotal role of epialleles arising from MITE insertions in governing the timing of flowering in maize.

The ZmNAC111 gene, which encodes a NAC transcription factor involved in plant development and stress responses, is another example of MITE-induced epigenetic regulation in maize. An 82 bp *Tc1/Mariner* MITE insertion located within the ZmNAC111 promoter region is significantly associated with drought tolerance [45]. This MITE insertion is specific to drought-sensitive lines and triggers the repression of ZmNAC111 expression through DNA methylation and H3K9me2 deposition. Over-expression of ZmNAC111 in *Arabidopsis* and maize transgenic lines enhances drought tolerance by regulating stomatal movement and the expression of genes that respond to drought.

In apple (*Malus domestica*), MdRFNR1 is a positive regulator of drought tolerance via modulation of the redox system. *MdRFNR1* encodes an NADP + oxidoreductase that is involved in converting NADPH to NADP +, thereby promoting ROS reduction [52]. A specific allele of this gene (*MdRFNR1-1*) harbours a 430 bp *PIF/Harbinger* MITE insertion within its promoter region. This allele is methylated and drought-responsive when compared to *MdRFNR1-2*, an allele devoid of the MITE insertion. The methylated, MITE-containing allele is targeted by anti-silencing factors, such as SUVH and DNAJ proteins, which enable drought-induced *MdRFNR1-1* expression. This example illustrates how MITE polymorphism insertions (MIPS) can influence transcript levels under drought stress conditions.

In rice, tillering is an important agronomic trait that impacts both structure and grain production, and is controlled by the RdDM pathway [73]. Under normal growth conditions, OsMIR156d/j genes induce tillering in rice and are repressed by CHH methylation on the two MITEs present in their respective promoter regions. Conversely, DWARF14, a gene whose expression inhibits tillering, is activated by CHH methylation on a downstream MITE, indicating that tillering in rice plants depends on the RdDM-dependent methylation status of MITEs. This knowledge could be used to develop markers for variety selection in this species.

#### MITEs promote alternative splicing

As MITEs preferentially insert into gene-rich regions, their insertions can also modulate alternative splicing mechanisms by introducing novel splice site sequences or by disrupting the splicing patterns of existing splice sites within the gene, resulting in a frameshift or truncated RNA, for example. This mechanism is illustrated in the following section.

In the mulberry (*Morus notabilis*), MITEs represent 14% of the genome [72]. The association between MITEs and differential alternative splicing in various tissue types revealed that alternative 5′ and 3′ splicing sites were the primary MITE-associated splicing variants. Exonisation of MITEs, or MITEs involved in alternative splicing, was predominantly found in flowers, illustrating the significant role that MITEs may play in gene regulation via splicing mechanisms in this species.

In the carrot genome, *hAT*-like MITEs are commonly found in gene regions and are co-transcribed with genes. They are also involved in forming new splicing variants in genes that harbour insertions. Similarly, genes for which differential exon usage has been reported are also enriched in *hAT*-like MITEs [44]. For example, MITE-containing isoforms were predominant for two genes (LOC108201852 and LOC108220652). The non-MITE-containing isoform of LOC108201852 is only expressed in bracts, whereas the MITE-containing isoform is highly expressed in bracts, flowers, roots, and seeds. The MITE-containing isoform of the LOC108220652 gene is expressed in all tissues, with the highest expression reported in bracts, open flowers, leaves, hypocotyls, phloem and xylem. In contrast, the non-MITE-containing isoform is only expressed in callus and leaves. Thus, MITEs contribute to the accumulation of new diversity in carrots by altering transcripts and directing the spatiotemporal regulation of gene expression.

#### MITEs induce mutations

Upon their new insertions, MITEs can induce mutations and loss of function. These mutations can have impacts on developmental traits as illustrated below.

The evolution of floral diversity among flowering species relies on the structures of petals and sepals. Master regulators such as *APETALA1 (AP1), APETALA3 (AP3), PISTILLATA (PI)* and *SEPALLATA1–4 (SEP1-4)* are known to be involved in flower development. In a natural apetalous *Nigella damascena* mutant, the petals transform into sepals, resulting in petal loss and the appearance of double layers of sepals. Gene expression analysis and genomic sequence investigation revealed that AP3-3 expression, a paralogue of AP3 which is usually expressed in petals, has been inactivated by a 253 bp MITE insertion in the second intron [76]. Actually, the MITE insertion does not prevent AP3-3 expression as the gene is still transcribed in the apetalous homozygous mutants but does not produce functional proteins [15]. In heterozygous plants, both allele are expressed, with the wild-type allele leading to production of functional proteins. This suggests the presence of a self-maintenance regulatory loop controlling AP3-3 expression. In the homozygous plants, the loop is disrupted with the absence of protein whereas in the heterozygous plants, the proteins produced by the functional allele seem to be sufficient to maintain this loop. Interestingly, in several other lineages within the *Ranunculaceae* family, the loss of petals has been associated with the inactivation of the AP3-3 gene through various genomic mechanisms, such as complete gene loss, partial deletion of the coding or promoter region, or MITE insertion, as observed in *Nigella damascena* [76].

In the *Rosaceae* family, self-incompatibility is determined by the interaction between pistil-specific RNases and pollen F-box proteins. This S-locus genetic control is further modulated by auxiliary factors known as modifier genes. In the apricot species *Prunus armeniaca*, self-compatible cultivars have a 358 bp *Mutator* MITE insertion named *FaSt* (*Falling Stone*) within the modifier gene ParMDO [31, 50]. This gene encodes an oxidoreductase protein and the MITE insertion results in the formation of a truncated protein which could potentially induce a loss of self-incompatibility.

#### MITEs mediate structural variants

There is also evidence that MITE insertions can facilitate complex genomic rearrangements. MITE copies can lead to chromosomal inversions, translocations or other structural variations through recombination, as presented in this section.

In *Brassica napus*, self-pollination is governed by the opening and closing behaviour of the petals. A genetic inversion spanning around 30 kb was identified through a comparative analysis between self-pollinated and cross-fertilised accessions (Fig. 2E). This inversion resulted in a reciprocal exchange of tissue-specific gene promoters between BnaC03.EFO1, which governs early flowering, and BnaC03.FBA, which is associated with F-box proteins and cleistogamy (Fig. 2E; [66]). The presence of two 327 bp *PIF/Harbinger* MITE insertions was detected at each breakpoint of this inversion, indicating the potential involvement of this MITE element in mediating the structural variant.

In angiosperms, the organisation of biosynthetic gene clusters, such as those involved in the production of terpenes, often arises through processes such as gene duplication, neofunctionalisation, and genomic rearrangement [54]. Boutanaev and Osbourn [5] show that MITEs are enriched around clustered terpene synthase and cytochrome P450 genes in the eudicots plants *Aquilegia coerulea* and *Kalanchoe fedtschenkoi* but not in the monocot *Amborella trichopoda*. They suggest that MITE could be driving the formation of these gene clusters by promoting recombination and may also contribute to their regulation as they are overrepresented near genes.

#### Transposition in action

MITEs are predominantly in a dormant state, but can exhibit spontaneous activation in response to various environmental fluctuations, genomic alterations within the host organism, or following hybridisation, tissue culture, or mutagenesis. Very few MITE bursts have been documented so far, however, and some of these are described here.

In rice, the *PIF/Harbinger mPing* was the first active MITE to be well described (Kikushi et al., 2003; [36, 37]). While inactive in most rice cultivars, *mPing* becomes active under specific environmental stresses, such as tissue culture or irradiation, and has been observed to burst naturally in 4 temperate japonica rice accessions. Despite lacking transposase-encoding sequences, the mobility of *mPing* is facilitated *in trans* by other autonomous transposons, such as *Ping* and *Pong*, which act as a source of transposases [12]. Notably, in specific rice accessions such as EG4 and AG123, *mPing* insertions modulate the expression of adjacent genes under cold stress conditions, thereby rewiring regulatory networks (see Fig. 2F and Table 1, [51]). The lack of coding sequences shared between *mPing* and the autonomous *Ping* may explain why *Ping* has not undergone epigenetic silencing. Additionally, the AT-rich insertion site of *mPing* may prevent insertion into GC-rich exons [12].

**Table 1:** Major plant MITEs shown to be actively transposing

MITE name	Superfamily	Species	Condition	Publication
*mPing*	*PIF/Harbinger*	*Oryza sativa*	irradiation/tissue culture/RILs	[12, 36–38]
*nDart1*	*hAT*	*Oryza sativa*	tissue culture	[48]
*dTok*	*hAT*	*Oryza sativa*	tissue culture	[34]
*nDaiZ*	*hAT*	*Oryza sativa*	tissue culture	[18]
*mJing*	*PIF/Harbinger*	*Oryza sativa*	tissue culture	[17]
*mGing*	*PIF/Harbinger*	*Oryza sativa*	irradiation	[64]
*dTstu1*	*Tc1/Mariner*	*Solanum tuberosum*	tissue culture	[47]
*AhMITE1*	*-*	*Arachis hypogaea*	irradiation/tissue culture/EMS	[27]
*PTE-1; PTE-2*	*Tc1/Mariner*	*Brassica rapa*	transformation	[35]

The mobility of *mPing* was studied in a population of rice RILs (recombinant inbred lines) over ten generations. A total of 87,450 *mPing* insertions were identified among the 272 RILs, corresponding to 16,914 *mPing* loci [11]. As most insertions were non-parental, they Likely occurred during the establishment of the recombinant Lines. The authors also identified 742 excision patterns within the RILs at 177 parental loci, demonstrating the ability of MITEs to excise from a site. Furthermore, the authors observed a strong correlation between *Ping* copy number and the frequency of new *mPing* insertions across the RILs. Thus, the activity and unsilenced state of *Ping* elements seem to control *mPing* transposition.

Following *mPing*, several additional active MITEs, including *nDart1*, *dTok* and *nDaiZ* (all members of the *hAT* superfamily), have been identified as being activated by tissue culture in rice (see Table 1; [18, 34, 48]). MITEs belonging to the *PIF/Harbinger* family, such as *mJing* and *mGing*, have also been shown to activate under stressful conditions, including γ-ray irradiation and tissue culture (Table 1, [17, 64]).

Tissue culture-activated MITEs have also been detected in potato. In the #72,218 potato variety, loss of expression of a flavonoid hydroxylase involved in anthocyanin synthesis is due to an inserted *Stowaway* MITE named *dTstu1* in the first exon of the gene. In the purple-skinned JKP variety, a somaclonal variant obtained from #72,218 protoplast culture, *dTstu1* is absent from the gene. This excision demonstrates recent activity. The reversion of the gene could explain the purple colour of JKP tubers (Table 1, [47]). Additionally, a MITE-display analysis revealed the de novo insertion of another related MITE in these somaclonal variants.

In the cultivated peanut (*Arachis hypogaea*), *AhMITE1* is an active 242 bp MITE. Following EMS mutagenesis of the Dharwad Early Runner cultivar, several Late Leaf Spot (LLS)-resistant mutants were isolated. These mutations were due to an *AhMITE1* insertion. Interestingly, the mutants reverted spontaneously through *AhMITE1* excision (Table 1, [28]).

Finally, two MITEs, *PTE-1* and *PTE-2*, from the *Tourist* and *Stowaway* families, respectively, were found to be active in transgenic *Brassica rapa* lines during the transformation process (Table 1, [35]).

#### Future prospects

- MITEs in pangenomes: Thanks to pangenomic studies, new functional roles for MITEs have been identified. These studies, coupled with SV-GWAS, have identified causal structural variants (SVs) associated with important plant phenotypes in species such as *Brassica oleracea* and poplar [39, 60]. As MITEs are not always properly annotated in genome assemblies, it is likely that many of the short SVs identified actually correspond to MITEs that have not yet been annotated. These large-scale studies will therefore help to clarify the functional role of MITEs.
- MIPs-GWAS: Systematic identification of MITEs insertion polymorphisms (MIPs) enables comparative analysis of MITE-mediated polymorphisms in different individuals or populations [9, 62]. MIPs have the potential to serve as markers that can elucidate the influence of MITEs on plant growth and adaptation through MIPs-GWAS analyses. MIPs could be used as markers in plant breeding programmes and biodiversity conservation plans [57, 59, 62].
- MITEs impact on gene expression: Long reads from new RNA-seq techniques such as Oxford Nanopore Direct RNA Sequencing are likely to extend our understanding of the impact of MITEs on gene expression [3], for instance by sequencing possible MITE-containing full-length transcripts.
- MITEs as precise gene targeting vectors: Recently, Slotkin’s laboratory has engineered MITEs for biotechnological purposes [41]. They fused a rice *Pong* transposase to Cas9 or Cas12a nucleases and used guide RNAs corresponding to the desired target loci to precisely insert transgenes into *Arabidopsis thaliana*. This toolkit has also been successfully transferred to soybean [41].

## Conclusion

MITEs are unusual among transposable elements due to their small size and non-autonomous nature. They are capable of modulating gene expression through their tendency to insert near genes or their regulatory regions. They are not usually transcribed, and their mobility depends on their autonomous counterparts, which encode transposases. As MITEs share Limited sequence homology with these autonomous copies, the effect of silencing could be Limited, which could explain their relative success in plant genomes. Depending on their insertion sites, MITEs can act as enhancers, repressors of gene expression. They can affect the methylation status of gene promoters by inducing the production of small RNAs. Such insertions can introduce novel transcription factor binding sites or induce changes in chromatin architecture, thereby affecting the transcriptional activity of neighboring genes, promoting alternative splicing and modulating 3D chromatin conformation. MITEs may therefore facilitate plant development, adaptability and evolution. Despite their limited representation in plant genomes, MITEs play a significant role in these processes, and the rapid progress of the sequencing era promises to further elucidate their centrality in plant genome evolution.

## Data Availability

No datasets were generated or analysed during the current study.
